# ISCCO: a deep learning feature extraction-based strategy framework for dynamic minimization of supply chain transportation cost losses

**DOI:** 10.7717/peerj-cs.2537

**Published:** 2024-12-12

**Authors:** Yangyan Li, Tingting Chen

**Affiliations:** 1 School of Accounting, Xijing University, Xi ’an, Shaanxi, China; 2 Department of Basic Faulty, Engineering University of PAP, Xi ’an, Shaanxi, China

**Keywords:** Deep learning, Transportation cost optimization, Pre-shipment policies, Data-Driven decisions, Intelligent supply chain cost optimization

## Abstract

With the rapid expansion of global e-commerce, effectively managing supply chains and optimizing transportation costs has become a key challenge for businesses. This research proposed a new framework named Intelligent Supply Chain Cost Optimization (ISCCO). ISCCO integrates deep learning with advanced optimization algorithms. It focuses on minimizing transportation costs by accurately predicting customer behavior and dynamically allocating goods. ISCCO significantly enhanced supply chain efficiency by implementing an innovative customer segmentation system. This system combines autoencoders with random forests to categorize customers based on their sensitivity to discounts and likelihood of cancellations. Additionally, ISCCO optimized goods allocation using a genetic algorithm enhanced integer linear programming model. By integrating real-time demand data, ISCCO dynamically adjusts the allocation of resources to minimize transportation inefficiencies. Experimental results show that this framework increased the accuracy of user classification from 50% to 95.73%, and reduced the model loss value from 0.75 to 0.2. Furthermore, the framework significantly reduced order cancellation rates in practical applications by adjusting pre-shipment policies, thereby optimizing profits and customer satisfaction. Specifically, when the pre-shipment ratio was 25%, the optimized profit was approximately 7.5% higher than the actual profit, and the order cancellation rate was reduced from a baseline of 50.79% to 41.39%. These data confirm that the ISCCO framework enhances logistics distribution efficiency. It also improves transparency and responsiveness across the supply chain through precise data-driven decisions. This achieves maximum cost-effectiveness.

## Introduction

### Background

With the rapid growth of global e-commerce, the efficiency and accuracy of order fulfillment—the process of completing and delivering customer orders—have become key indicators of business competitiveness. Optimizing inventory allocation and order processing to reduce cost losses due to delivery delays is a current hot topic of research. According to surveys, up to 69% of consumers state that if the goods purchased are not delivered within the promised two days, their future purchasing intention would significantly decrease (Lindner, 2024). Such delivery delays not only harm customer satisfaction but also increase the operational costs for businesses. Research shows that 72.5% of customers might not recommend a retailer after poor delivery services. Therefore, optimizing the delivery experience is a crucial strategy for reducing operational costs and enhancing market competitiveness (Circuit, 2021). Additionally, data indicates that 77% of respondents rated their delivery experience at least 8 out of 10 in the past six months (Winters, 2024) proving the importance of effective logistics services in fostering continued customer purchasing behaviors.

Despite the critical role of optimizing logistics and the delivery process in reducing cost losses, practical challenges persist. In an e-commerce environment, the large and highly unstable order volume—especially during periods of sudden demand spikes or drops—poses a significant challenge for supply chain management (Umar & Wilson, 2024). Supply chain systems need to process vast amounts of frequently updated data in real-time. Traditional data processing methods often fail to meet the high-efficiency and accuracy demands of these dynamic environments (Mirbagheri, 2023). Modern e-commerce platforms use data mining to collect multi-dimensional data on consumer behavior, which traditional supply chain models often overlook. This limitation prevents traditional models from achieving personalized resource allocation and optimization based on deeper insights into customer behavior (Zhang et al., 2022). Complex resource configurations, such as inventory management and transportation scheduling, require precise and real-time decision-making support (Wang & Huang, 2022). The pressure to ensure on-time delivery makes accurate prediction and efficient handling of shipping strategies both critical and challenging.

### Related work

In recent years, the application of machine learning and deep learning in supply chain management and project cost prediction has garnered increasing attention. However, existing studies still exhibit certain limitations in terms of feature extraction and nonlinear feature processing. Bodendorf, Merkl & Franke (2021) explored the use of machine learning models to estimate procurement part costs in supply chain management. However, this study did not incorporate advanced feature extraction methods, such as deep learning, which are crucial for handling complex data structures and improving prediction accuracy. Similarly, Inan, Narbaev & Hazir (2022) applied a long short-term memory (LSTM) model for project cost prediction but did not fully utilize nonlinear feature processing techniques, which limited the accuracy of the prediction model. Another study by Abed, Hasan & Zehawi (2022) assessed machine learning and deep learning applications in construction cost prediction but highlighted the challenge of feature extraction and handling dynamic data, which restricted the model’s effectiveness in rapidly changing environments.

An increasing number of researchers have focused on optimizing logistics and supply chain processes to reduce costs. Several machine learning models have been proposed in the literature for cost prediction, order management, and logistics optimization (Chong et al., 2024; Yong-Cai, 2024; Cho et al., 2024). Table 1 summarizes related works and highlights their limitations. These works primarily focus on predictive modeling and cost estimation but lack an integrated approach to customer behavior segmentation, real-time dynamic resource allocation, and optimization based on multiple data features.

**Table 1:** Comparative analysis of literature.

**Author**	**Application scenario**	**Research content**	**Potential shortcomings**
Inan, Narbaev & Hazir (2022)	Project cost prediction	Machine learning model based on Long Short-Term Memory for project cost prediction	Method does not fully utilize nonlinear feature processing techniques, limiting the accuracy of the prediction model
Bodendorf, Merkl & Franke (2021)	Cost estimation in supply chain management	Estimation of costs for procurement parts using machine learning	Lack of deep learning and feature extraction methods for complex data structures, affecting the efficiency of cost prediction
Abed, Hasan & Zehawi (2022)	Construction cost prediction	Assessment of machine learning and deep learning applications in construction cost prediction	Model lacks capability in feature extraction and handling dynamic data sets
Wang & Qiu (2023)	Impact of AI on labor costs	Exploration of AI applications in labor cost decision-making	Failure to effectively integrate multidimensional data processing and optimization algorithms, limiting dynamic adaptability in decision-making
Fernandez-Revuelta Perez & Romero Blasco (2022)	Cost estimation decision-making	Use of data science methods for cost estimation	Lack of effective algorithm optimization and nonlinear data handling strategies, impacting the accuracy of the cost model
Uddin et al. (2023)	Project management	Integration of machine learning and network analysis to simulate project cost, time, and quality performance	Lack of efficient data feature learning and model tuning mechanisms in project management models
Mahdi et al. (2021)	Software project management	Discussion of machine learning applications in software project management	Insufficient handling of variable and complex data features in software projects, limiting model generalization capability
Dang-Trinh et al. (2023)	Factory construction cost estimation	Use of various machine learning models to predict initial costs of factory construction	Method lacks adaptability and efficiency in handling large-scale and complex data

While these studies have made significant contributions to cost estimation in specific domains, they generally fall short of providing an adaptable, real-time solution for supply chain optimization. Additionally, the majority of the models fail to integrate multidimensional data sources, which limits their ability to provide comprehensive insights into customer behavior and resource allocation. This gap underscores the need for a more integrated and dynamic approach to supply chain cost optimization.

### Our contributions

**Figure 1:**
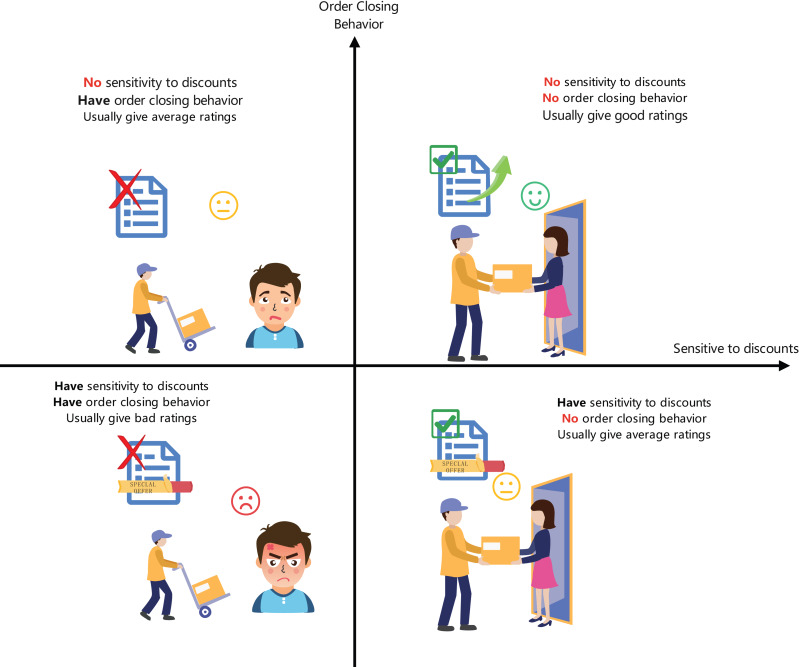
Four dimensions of user categorization.

• **Multi-dimensional classification of customer behavior:** This study introduces an innovative classification method combining autoencoders with random forests to accurately predict users’ sensitivity to discounts and the probability of order cancellation, as shown in Fig. 1. This method, by learning users’ purchasing behaviors and response patterns, enhances the accuracy and adaptability of the classification.
• **Intelligent goods allocation strategy:** This research develops a parallel genetic algorithm-enhanced integer linear programming model (GA-ILP) specifically for optimizing goods allocation in pre-shipment, effectively reducing logistics costs caused by order cancellations.
• **Resource optimization and algorithm adaptability:** Utilizing parallel genetic algorithms to dynamically optimize the parameters of the goods allocation model, adjusting algorithm control parameters such as crossover rate and mutation rate based on real-time data, enhances the adaptability and flexibility of the algorithm.

## The ISCCO Framework

### Optimisation problem of supply chain cost control

Firstly, customers are categorized based on their sensitivity to product discounts and their tendency to cancel orders. We define the customer classification function *C* which outputs customer types across four dimensions:

(1) \begin{eqnarray*}C(X)=\text{Category}(X)\end{eqnarray*}

where *X* represents the dataset containing experimental features. Subsequently, the algorithm optimizes the shipping strategy for goods based on customer type and order data. We define the goods allocation strategy function *S*, aiming to minimize the transportation cost losses *L* incurred from order cancellations:

(2) \begin{eqnarray*}L=\sum _{i=1}^{N}{w}_{i}\cdot {c}_{i}\cdot \mathbf{1}({o}_{i}=\text{canceled})\end{eqnarray*}

where *w*_*i*_ represents the weight of importance of the *i*-th order, *c*_*i*_ represents the cost loss due to cancellation, and **1** is an indicator function that takes the value 1 when order *o*_*i*_ is canceled, otherwise 0. The goods allocation strategy *S* predicts and minimizes cost losses *L* based on the customer type *C*(*X*) and other order features *Y*:

(3) \begin{eqnarray*}\hat {L}=S(C(X),Y;\theta )\end{eqnarray*}

where *θ* represents model parameters. The model’s training objective is to minimize the mean squared error between the predicted and actual cost losses:

(4) \begin{eqnarray*}\min _{\theta } \frac{1}{N} \sum _{i=1}^{N}{ \left( {L}_{i}-{\hat {L}}_{i} \right) }^{2}.\end{eqnarray*}

Here, *L*_*i*_ and ${\hat {L}}_{i}$ are the actual and predicted cost losses for the *i*-th sample, respectively. The model needs to capture nonlinear relationships and interactions across multiple dimensions including customer discount sensitivity, cancellation tendency, order delay risk, geographical location, and order value.

Problem 1 *The core problem is how to design an optimization function *S* that effectively handles and adapts to multidimensional, nonlinear feature interactions and minimizes the above mean squared error to accurately predict and reduce the transportation cost losses due to order cancellations.*

(5) \begin{eqnarray*}\min _{S}&  \mathbb{E} \left[ { \left( L-S(C(X),Y) \right) }^{2} \right] .\end{eqnarray*}

\begin{eqnarray*}subject~to&  S~must~adapt~to~high-dimensional,~nonlinear~feature~interactions. \end{eqnarray*}

### Customer dimension classification

#### Introducing deep learning feature extraction with random forest: achieving efficient customer classification

**Figure 2:**
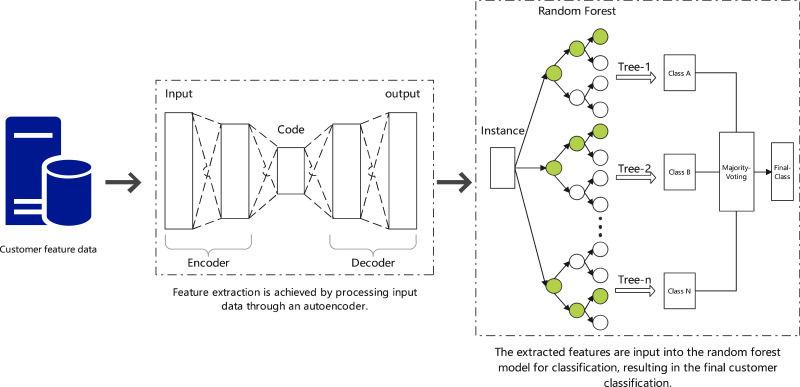
Customer classification using autoencoders and random forests.

• Traditional supply chain management often overlooks the multidimensional characteristics of customer behavior, limiting the efficiency and precision of resource allocation (Capó, Pérez & Lozano, 2020). Traditional algorithms also fail to capture this type of nonlinear similarity, restricting their application in this scenario (Baldassi, 2022; Ay et al., 2023; Ikotun et al., 2023).
• This study proposes a new customer classification strategy by combining deep learning with random forests, which not only handles complex nonlinear features but also effectively extracts key information through autoencoders, greatly enhancing the accuracy and generalization capability of the classification.
• As shown in Fig. 2, this process combines autoencoders for feature extraction and random forests for customer classification. The autoencoder compresses the input data into key features, which are then classified by the random forest model. The model’s performance is optimized using cross-validation and grid search, aiming to improve customer segmentation and reduce transportation costs through more accurate pre-shipment decisions.

#### Implementing deep learning feature extraction with random forest: achieving efficient customer classification

In the context of supply chain cost control, we propose a method combining autoencoders with random forests for customer classification. Autoencoders are used to process input features and extract key nonlinear information. The encoding process can be extended by adding multiple layers and including regularization terms, represented by the equation:

(6) \begin{eqnarray*}h={f}_{3} \left( {W}^{(3)}{f}_{2} \left( {W}^{(2)}{f}_{1} \left( {W}^{(1)}x+{b}^{(1)} \right) +{b}^{(2)}+\lambda \parallel {W}^{(2)}{\parallel }_{2} \right) +{b}^{(3)} \right) .\end{eqnarray*}

The decoding process is specifically expressed by the following equation:

(7) \begin{eqnarray*}{x}^{{^{\prime}}}={g}_{3} \left( {{W}^{{^{\prime}}}}^{(3)}{g}_{2} \left( {{W}^{{^{\prime}}}}^{(2)}{g}_{1} \left( {{W}^{{^{\prime}}}}^{(1)}h+{{b}^{{^{\prime}}}}^{(1)} \right) +{{b}^{{^{\prime}}}}^{(2)}+\lambda \parallel {{W}^{{^{\prime}}}}^{(2)}{\parallel }_{2} \right) +{{b}^{{^{\prime}}}}^{(3)} \right) .\end{eqnarray*}

In these equations, *x* is the input vector, *W*^(*i*)^ and *W*′^(*i*)^ are the weight matrices for encoding and decoding stages, respectively, *b*^(*i*)^ and *b*′^(*i*)^ are bias vectors, *f*_*i*_ and *g*_*i*_ are activation functions, *λ* is a regularization coefficient, *h*^(*i*)^ and *x*^(*i*)^ represent outputs at each layer. Subsequently, using the compressed features *h* obtained from the autoencoder, the classification result of the random forest model *C*(*x*) can be defined by:

(8) \begin{eqnarray*}C(x)=\sum _{i=1}^{N}{w}_{i}\cdot {c}_{i}(h)+\gamma \cdot \text{Var}({c}_{i}(h)).\end{eqnarray*}

Here, *N* is the number of decision trees, *c*_*i*_ is the classification result of the *i*-th tree on the compressed feature *h*, *w*_*i*_ represents the weight of the *i*-th tree, reflecting its importance in the overall classification, *γ* is an adjustment factor, Var(*c*_*i*_(*h*)) measures the variance of the classification results, used to assess the uncertainty of the results. To ensure model generalization and prevent overfitting, we employ cross-validation techniques and optimize model hyperparameters through grid search. The overall performance of the model is evaluated by the weighted average of various metrics under cross-validation, as expressed below:

(9) \begin{eqnarray*}J=\sum _{i=1}^{n}{\alpha }_{i} \left( \frac{1}{k} \sum _{j=1}^{k}{\text{Metric}}_{i}({M}_{j},{D}_{\text{val},j}) \right) .\end{eqnarray*}

Here, *k* is the number of folds in cross-validation, *M*_*j*_ is the model trained on the *j*-th fold, *D*_val,*j*_ is the corresponding validation set, Metric_*i*_ represents the *i*-th evaluation metric, *α*_*i*_ is the weight of the corresponding evaluation metric.

Theorem 1 **Optimization of customer classification**

*By combining autoencoders with random forests, the accuracy of classification across different customer dimensions can be significantly enhanced. There exists an optimal set of parameters Θ^∗^, *W*^∗^, *b*^∗^ that optimizes overall model performance:*

(10) \begin{eqnarray*}{\Theta }^{\ast },{W}^{\ast },{b}^{\ast }=\arg \nolimits \min _{\Theta ,W,b} \left\{ J(\Theta ,W,b)-\lambda \cdot \sum _{i=1}^{N}{\alpha }_{i}\cdot \log \nolimits \frac{p({y}_{i}{|}\Theta ,W,b,{x}_{i})}{p({y}_{i}{|}{x}_{i})} \right\} .\end{eqnarray*}

*Here, *J*(Θ, *W*, *b*) is the model performance evaluation function obtained through cross-validation, *λ* and *α*_*i*_ are adjustment coefficients, where *α*_*i*_ represents the importance of the *i*-th data point, *p*(*y*_*i*_|*x*_*i*_) is the probability of category *y*_*i*_ given the input *x*_*i*_.*

Corollary 1 **Enhancement of cost efficiency**

*By reasonably allocating goods that can be shipped in advance based on customer classification, the transportation cost losses caused by order cancellations can be effectively reduced. By applying the optimized classification model, goods pre-shipped are allocated more precisely to users across various dimensions, thereby maximizing cost efficiency:*

(11) \begin{eqnarray*}{\Theta }_{eff}^{\ast }=\arg \nolimits \min _{\Theta } \left\{ J(\Theta )+\gamma \cdot \text{Var}(C(\Theta )) \right\} .\end{eqnarray*}

*Here, *J*(Θ) is the cross-validation performance evaluation based on the model, *γ* is an adjustment factor, *Var*(*C*(Θ)) indicates the variance in customer dimension classification using the random forest model, aimed at measuring the stability and accuracy of the classification results.*

The specific validation process is shown in the appendix.

### Minimization of transportation cost losses in goods allocation strategy

#### Enhanced integer linear programming with genetic algorithm: implementing a cost loss minimization strategy for goods allocation

**Figure 3:**
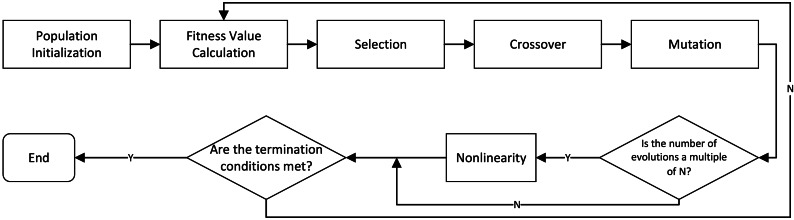
Process of parallel genetic algorithm optimizing integer linear programming model.

• Many traditional models rely on a static decision-making environment, assuming that data do not change over time. This results in significant efficiency reductions when the model fails to adapt to new requirements once environmental changes occur (Alfaro, Valencia & Vargas, 2023; Prerna & Sharma, 2022). Moreover, traditional models often struggle to effectively handle uncertainties in the supply chain, such as demand fluctuations and supply disruptions, because they lack mechanisms for flexible decision-making (Dupin, 2022).
• Our proposed integer linear programming model enhanced with a parallel genetic algorithm (GA-ILP) combines the global search capabilities of genetic algorithms with the precise optimization of integer linear programming to achieve efficient optimization of goods allocation in the supply chain. This algorithm not only optimizes total costs resulting from order cancellations or delays but also significantly enhances computational efficiency through parallel processing techniques.
• Figure 3 shows a process where a parallel genetic algorithm (GA-ILP) optimizes an Integer Linear Programming model. It starts by initializing a population, then calculates fitness values. The algorithm evolves solutions through selection, crossover, and mutation. If termination conditions are met, the process ends; otherwise, it continues iterating until the optimal solution is found.

#### Enhanced integer linear programming with parallel genetic algorithm (GA-ILP)

Research proposes an algorithm for an enhanced integer linear programming (ILP) model using a parallel genetic algorithm (GA-ILP), aimed at optimizing goods allocation to reduce order cancellation rates and transportation costs. The model achieves precise optimization of goods distribution. Initially, an ILP model is constructed with the objective of minimizing the total cost incurred from order cancellations or delays, expressed as:

(12) \begin{eqnarray*}\text{Minimize} Z=\sum _{i=1}^{n}\sum _{j=1}^{m} \left( {c}_{ij}\cdot {x}_{ij}+{p}_{ij}\cdot (1-{x}_{ij}) \right) +\lambda \sum _{i=1}^{n}\sum _{j=1}^{m}{s}_{ij}\cdot {x}_{ij}\end{eqnarray*}

Here, *n* represents the number of orders, *m* represents the types of goods. *c*_*ij*_ is the cost if item *j* in order *i* is canceled, *p*_*ij*_ is the penalty if item *j* in order *i* is delayed. *x*_*ij*_ is a binary decision variable indicating whether to allocate item *j* to order *i*, *s*_*ij*_ is the storage or holding cost, and *λ* is a cost adjustment factor.

(13) \begin{eqnarray*}\sum _{i=1}^{n} \left( {x}_{ij}+ \frac{{t}_{ij}\cdot {x}_{ij}}{{T}_{i}} \right) \leq {q}_{j}+ \frac{1}{m} \sum _{k=1}^{m} \frac{{T}_{k}}{{t}_{kj}}  \forall j\end{eqnarray*}

(14) \begin{eqnarray*}\sum _{j=1}^{m} \left( {x}_{ij}+ \frac{{p}_{i}\cdot {x}_{ij}}{{P}_{\text{min},j}} \right) =1+ \frac{1}{n} \sum _{k=1}^{n} \frac{{P}_{\text{min},k}}{{p}_{k}}  \forall i.\end{eqnarray*}

Equation (13) integrates inventory quantities with delivery time constraints. Equation (14) integrates the requirement that each order must be allocated at least one type of item, considering order priority to ensure high-priority orders are prioritized during goods allocation. Decision variable *x*_*ij*_ must not only meet integer conditions but also consider order fulfillment rates and real-time inventory adjustments. Thus, we introduce a continuous decision variable *y*_*ij*_, representing the probability of fulfilling order *i* with item *j*, influenced dynamically by inventory adjustments. The integer condition and fulfillment probability are formulated as:

(15) \begin{eqnarray*}{x}_{ij}\in \{ 0,1\} , {y}_{ij}={x}_{ij}\times (1-{e}^{-\lambda {t}_{ij}}) \forall i,\forall j\end{eqnarray*}

where *e*^−*λt*_*ij*_^ represents the decayed fulfillment probability due to the response time *t*_*ij*_, and *λ* is the decay coefficient. To solve this integer linear programming problem, we use a parallel genetic algorithm for optimization. The algorithm is described as:

(16) \begin{eqnarray*}\text{Parallel GA}(\Theta ,x,y) & =\text{arg}~\min _{x,y} \left\{ \alpha \cdot Z(\Theta ,x,y)+\beta \cdot \text{Var}(x,y)+\gamma \cdot \text{Stab}(x,y) \right\} & \text{subject to} &  {g}_{k}(x,y)\leq 0, {h}_{l}(x,y)=0 \forall k,\forall l.\end{eqnarray*}

Here, *α*, *β*, and *γ* are weighting factors, used to balance the importance of different objectives. *Z*(Θ, *x*, *y*) is the original cost function, Var(*x*, *y*) evaluates the variance of the solution for diversity assessment, and Stab(*x*, *y*) is a new stability metric, measuring the consistency of solutions across multiple runs. The constraints *g*_*k*_(*x*, *y*) ≤ 0 and *h*_*l*_(*x*, *y*) = 0 represent the model’s inequality and equality constraints, allowing more precise control over the feasibility of solutions. The mathematical description of the algorithm is:

(17) \begin{eqnarray*}\text{GA-ILP}(\Theta ,x) & =\text{arg}~\min _{x} \left\{ \alpha \cdot Z(\Theta ,x)+\beta \cdot \text{Var}(x)+\gamma \cdot \text{Stab}(x)+\delta \cdot \text{Cons}(x) \right\} & \text{subject to} &  {c}_{i}(x)\leq 0, \forall i\end{eqnarray*}

where Θ includes the control parameters of the genetic algorithm, the formula’s *α* controls the impact of the cost function *Z*(Θ, *x*). *β* adjusts the variance of the solution Var(*x*), measuring solution diversity. *γ* is the stability metric Stab(*x*), assessing consistency across multiple runs. *δ* is the constraint satisfaction metric Cons(*x*), quantifying the degree of constraint violation of the solution. *c*_*i*_(*x*) represents the problem’s constraints, ensuring the feasibility of the solution. The model not only provides a comprehensive optimization framework but also enhances the applicability and efficiency of the algorithm in practical applications through multi-objective and constraint management.

Theorem 2 **Optimizing integer linear programming with parallel genetic algorithm**

*There exists an optimal set of parameters Θ^∗^ that optimizes the model under given cost functions and constraints: Where *Z*(Θ, *x*, *y*) is the cost function, *Var*(*x*, *y*) represents the variance of the solution, *Stab*(*x*, *y*) is the stability of the solution.*

(18) \begin{eqnarray*}{\Theta }^{\ast }=\arg \nolimits \min _{\Theta } \left\{ Z(\Theta ,x,y)+\beta \cdot \text{Var}(x,y)+\gamma \cdot \text{Stab}(x,y) \right\} \end{eqnarray*}

Corollary 2 **Parameter adjustment and system performance enhancement**

*In the enhanced integer linear programming model with parallel genetic algorithm, optimizing parameters Θ can achieve higher system performance and cost efficiency: Where *α*, *β*, and *γ* are weighting factors, used to balance cost, variance, and stability.*

(19) \begin{eqnarray*}{\Theta }^{\ast }=\arg \nolimits \min _{\Theta } \left\{ \alpha \cdot Z(\Theta )+\beta \cdot \text{Var}(C(\Theta ))+\gamma \cdot \text{Stab}(C(\Theta )) \right\} \end{eqnarray*}

Details of the computations can be found in the appendix.

### ISCCO framework

#### Overall structure of the ISCCO framework

The core of the ISCCO framework is to efficiently categorize customers using deep learning and utilize this classification to optimize goods allocation strategies, minimizing transportation costs and order cancellation rates. The structure of the framework is as follows:

(20) \begin{eqnarray*}\text{ISCCO}(\Theta ,x,y) & =\arg \nolimits \min _{\Theta ,x,y} \left\{ \alpha \cdot J(\Theta ,x,y)+\beta \cdot \text{Cost}(x,y)+\gamma \cdot \text{Risk}(x,y) \right\} & \text{subject to} &  {g}_{k}(x,y)\leq 0, {h}_{l}(x,y)=0 \forall k,\forall l\end{eqnarray*}

where *α*, *β*, and *γ* are weighting factors used to balance the importance of different objectives. *J*(Θ, *x*, *y*) is the performance evaluation function based on customer classification, Cost(*x*, *y*) represents the total cost incurred from goods allocation, and Risk(*x*, *y*) represents the risk arising from order cancellations or delays. In the ISCCO framework, the optimization of customer classification and goods allocation is carried out interactively. This process is represented by the following mathematical model:

(21) \begin{eqnarray*}{\Theta }_{ISCCO}^{\ast },{x}^{\ast },{y}^{\ast }=\arg \nolimits \min _{\Theta ,x,y} \left\{ J(\Theta )+{\lambda }_{1}\cdot \text{Var}(C(\Theta ))+{\lambda }_{2}\cdot \text{Cost}(x,y)+{\lambda }_{3}\cdot \text{Risk}(x,y) \right\} \end{eqnarray*}

where *λ*_1_, *λ*_2_, and *λ*_3_ are adjustment coefficients used to balance the trade-offs among classification accuracy, cost efficiency, and risk management. *C*(Θ) represents the output of customer classification, Var(*C*(Θ)) is the variance of classification results, used to assess the stability of classifications. The ISCCO framework must satisfy a series of constraints, which can be represented as:

(22) \begin{eqnarray*}{g}_{k}(x,y)={ \left( \sum _{i=1}^{n}{x}_{ij}-{q}_{j} \right) }^{2}\leq 0, \forall j\end{eqnarray*}

(23) \begin{eqnarray*}{h}_{l}(x,y)= \left( \sum _{j=1}^{m}{x}_{ij}-1 \right) =0, \forall i\end{eqnarray*}

where *q*_*j*_ is the inventory level for the *j*th type of good, *x*_*ij*_ is the decision variable indicating whether good *j* is allocated to order *i*. Based on this, research focuses on minimizing transportation costs. Initially, users are divided into four dimensions based on their sensitivity to product discounts and propensity to cancel orders. This can be achieved through a random forest approach with deep learning feature extraction. The classification model *C* can be expressed as:

(24) \begin{eqnarray*}C(x)=\text{RF}(\text{AE}(x;W,b);\Theta )\end{eqnarray*}

where AE represents the autoencoder used for feature extraction, *W*, *b* are the network parameters, RF represents the random forest classifier, Θ is the parameters of the random forest. Based on the classification results from Eq. (24), we further define a goods allocation strategy. This strategy aims to minimize the transportation cost losses incurred from order cancellations. The goods allocation strategy *S* can be defined by the following expression:

(25) \begin{eqnarray*}S(C(x),Y;\theta )=\min \nolimits \left( \sum _{k=1}^{4}\sum _{i\in {D}_{k}}{w}_{i}\cdot {c}_{i}\cdot \mathbf{1}({o}_{i}=\text{cancel}) \right) \end{eqnarray*}

where *D*_*k*_ is the set of orders belonging to the *k*-th customer class, *w*_*i*_ is the weight of an order, *c*_*i*_ is the cost incurred from cancelling an order, **1** is an indicator function. Our goal is to minimize the total cost *L* by optimizing the goods allocation strategy *S*. This optimization problem can be represented as:

(26) \begin{eqnarray*}\min _{\theta } \left( \sum _{k=1}^{4}{L}_{k} \right) =\min _{\theta } \left( \sum _{k=1}^{4}\sum _{iin{D}_{k}}{w}_{i}\cdot {c}_{i}\cdot \mathbf{1}({o}_{i}=\text{cancel}). \right) \end{eqnarray*}

Here *L*_*k*_ represents the cost loss caused by the *k*-th class of users. Finally, the optimization of pre-shipped orders allocated to four different dimensions of users can be expressed using the following mathematical formula:

(27) \begin{eqnarray*}\min \nolimits L={w}_{1}\times {L}_{1}+{w}_{2}\times {L}_{2}+{w}_{3}\times {L}_{3}+{w}_{4}\times {L}_{4}\end{eqnarray*}

where *w*_*k*_ are weights for the *k*-th class of users, *L*_*k*_ is the predicted cost loss for that class of users, these weights may depend on the order cancellation rate of the class or other business strategy factors.

#### Algorithm pseudocode and complexity analysis


 
_______________________ 
 Algorithm 1: Optimized Supply Chain Cost Control Algorithm (IS- 
  CCO)                                                                                    _________ 
    Input:   Dataset X  with features on customer purchase 
           behavior and response patterns, Additional order 
           features Y , Initial parameters Θ 
    Output:   Optimized transportation costs L 
    // Feature extraction and customer classification 
 1  Function AutoEncoder(X): 
     2   Encode features to reduce dimensionality and extract 
   nonlinear patterns using Eq. 6 and Eq. 7; 
3   return encoded features h; 
 4  Function RandomForestClassifier(h, ΘRF): 
     5   Classify customers into four categories based on encoded 
   features and sensitivity to discounts and cancellation 
   likelihood using Eq. 8; 
6   return customer categories C(X); 
   // Goods distribution optimization 
 7  Function IntegerLinearProgramming(C(X), Y , ΘILP): 
     8   Optimize goods allocation by minimizing expected cost 
   losses from order cancellations using a genetic 
   algorithm-enhanced ILP model as per Eq. 12 to Eq. 17; 
9   return minimized cost function Z; 
10  Function CostStrategy(C(X), Y , ΘCS): 
     11   Distribute pre-shipped goods to different customer 
   categories to minimize cancellation-related costs using 
   Eq. 23 to Eq. 25; 
12   return updated cost L; 
13  h ← AutoEncoder(X); 
14  C(X)  ← RandomForestClassifier(h, ΘRF); 
15  Y  ← Prepare additional order features; 
16  Z  ← IntegerLinearProgramming(C(X), Y , ΘILP); 
17  L ← CostStrategy(C(X), Y , ΘCS); 
18  return Optimized transportation costs L;    


Considering time complexity, ISCCO is mainly influenced by the number of features, the number of trees, and decision variables, showing potential efficiency in applications with high data complexity. According to Algorithm 1 , the time complexity is $\mathcal{O}({n}^{2}+N&sdot; \log n+m)$, involving feature dimension reduction, customer classification, and goods distribution optimization. In terms of space complexity, the algorithm’s space complexity is $\mathcal{O}({n}^{2}+N&sdot; n+m)$, reflecting the space required to store weights for the autoencoder, the structure of the random forest, and the linear programming model.

Comparing the ISCCO framework with other models in the same field, as shown in Table 2, it is evident that the ISCCO framework has a time complexity of $\mathcal{O}({n}^{2}+N&sdot; \log n+m)$ and a space complexity of $\mathcal{O}({n}^{2}+N&sdot; n+m)$. Compared to the algorithm by Cao & Liu (2021) (time complexity $\mathcal{O}({n}^{3})$, space complexity $\mathcal{O}({n}^{2})$), the ISCCO framework can more efficiently utilize computing resources to reduce time costs when handling large-scale data. In terms of space complexity, the ISCCO framework’s complexity is $\mathcal{O}({n}^{2}+N&sdot; n+m)$, which is similar to the algorithm of Sun et al. (2020), but the ISCCO framework may be more efficient for large data sets. The increased space requirement is to support more complex data structures and caching mechanisms, thereby optimizing the execution efficiency and response time of the algorithm.

**Table 2:** Comparison of time and space complexities.

Algorithm	Time complexity	Space complexity
Belogaev et al. (2020)	$\mathcal{O}(m&sdot; \log m)$	$\mathcal{O}(m+c)$
Cao & Liu (2021)	$\mathcal{O}({n}^{3})$	$\mathcal{O}({n}^{2})$
Raskin et al. (2021)	Complex (iterative, non-linear)	$\mathcal{O}(m+c)$
Sun et al. (2020)	$\mathcal{O}({n}^{2}&sdot; L)$	$\mathcal{O}(L&sdot; {n}^{2})$
ISCCO Framework	$\mathcal{O}({n}^{2}+N&sdot; \log n+m)$	$\mathcal{O}({n}^{2}+N&sdot; n+m)$

## Experimental Results

### Dataset and experimental parameters introduction

**Dataset Description:** The dataset used in this study is named “DataCo SMART SUPPLY CHAIN FOR BIG DATA ANALYSIS”, which includes supply chain data utilized by DataCo Global (https://tianchi.aliyun.com/dataset/89959). The dataset encompasses activities across the entire supply chain from procurement, production to sales, and business distribution, containing both structured and unstructured data, allowing for supply chain-related data analysis. Our experimental parameters are set as shown in Table 3.

**Table 3:** Experimental parameters.

Parameter name	Parameter value	Parameter name	Parameter value
Dataset	DataCo smart supply chain	Number of training epochs	30
Autoencoder layers	3 (input layer, 2 hidden layers, output layer)	Neurons per Layer	Input 256, Hidden 150/300/150, Output 256
Autoencoder activation function	ELU	Autoencoder optimizer	Adam
Autoencoder Learning Rate	0.001	Autoencoder Batch Size	128
Autoencoder regularization	L1+L2, Parameter=0.05	Autoencoder iterations	50
Random forest number of trees	100	Random forest max depth	Unlimited
Random forest evaluation metrics	Precision, F1 score	Genetic algorithm population size	100
Genetic Algorithm Crossover Rate	0.8	Genetic Algorithm Mutation Rate	0.2
Data preprocessing	Standardization	Loss function	Mean squared error
**ANN parameters**			
ANN layers	4 (input layer, 2 hidden layers, output layer)	Neurons per Layer	Input 256, Hidden 128/64, Output 10
ANN activation function	ReLU	ANN optimizer	Adam
ANN learning rate	0.001	ANN batch size	64
**CNN parameters**			
CNN layers	5 (convolutional layers + fully connected layers)	Filters per Layer	32, 64, 128
CNN kernel size	3x3	CNN activation function	ReLU
CNN optimizer	Adam	CNN batch size	32
CNN learning rate	0.0001	Pooling type	Max pooling

**Figure 4:**
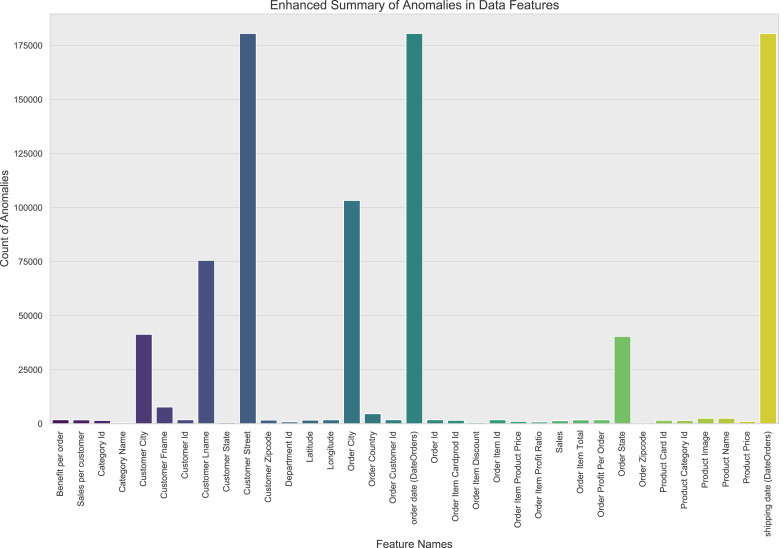
Information on anomalous data in the dataset.

In this study, through comprehensive outlier detection, it was found that some features involve sensitive information and were processed, resulting in generally anomalous values, as shown in Fig. 4. The dataset used for this research contains a total of 180,520 samples. The data was split into training and testing sets with an 80/20 ratio, resulting in 144,416 samples for training and 36,104 samples for testing. For those features where the proportion of outliers exceeds 90%, the choice was made to delete them directly, as their high proportion of outliers would severely disrupt the effectiveness of model training. For other features containing outliers, the study applied regression imputation methods to correct and fill in anomalous data points.

It should be noted that all the experimental results, are based on testing samples. This ensures that the reported performance metrics reflect the model’s ability to generalize to unseen data.

### Model performance analysis

**Figure 5:**
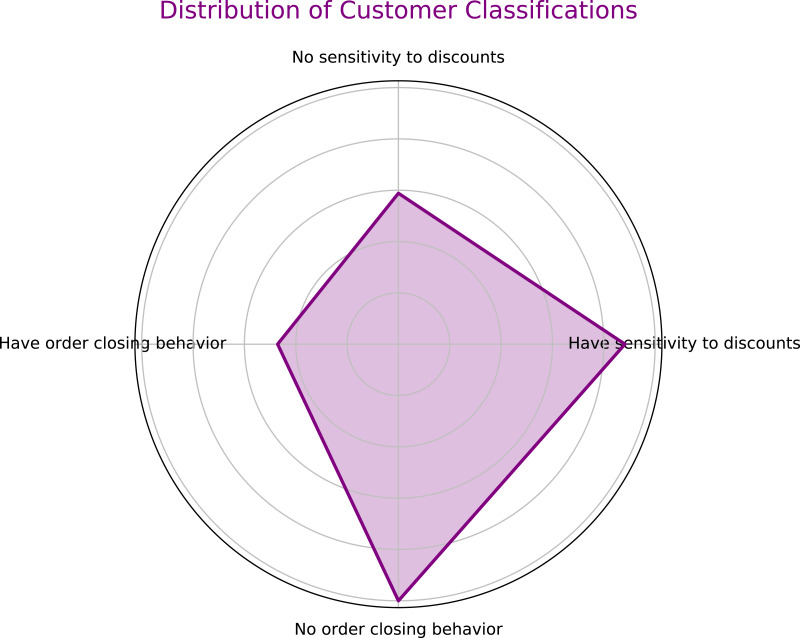
User classification results.

**Figure 6:**
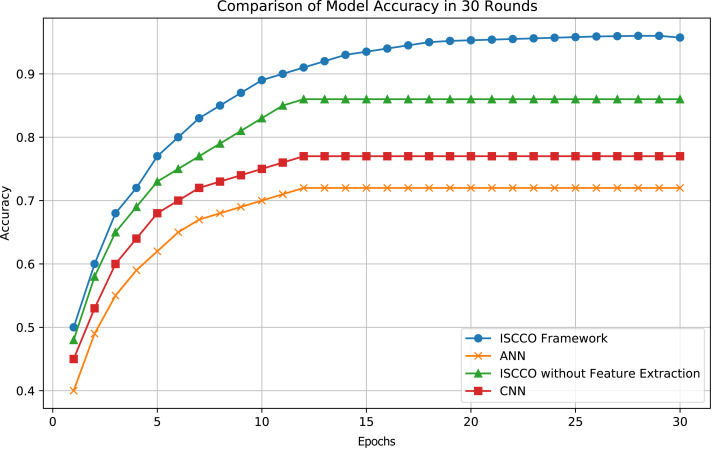
Model accuracy comparison.

The customer classification results of the model are shown in Fig. 5. Experiments demonstrate that the ISCCO framework surpasses other models in terms of accuracy and loss values, as shown in Fig. 6. Notably, in terms of accuracy, the ISCCO framework improved from 50% to 95.73%, while the artificial neural network (ANN) model, although improving from 40% to 85%, did not match the enhancement magnitude and final accuracy of the ISCCO framework. Similarly, the ISCCO version without feature extraction only improved from 48% to 86%, which is lower than the performance after introducing feature extraction, confirming the crucial role of feature extraction in enhancing model accuracy.

**Figure 7:**
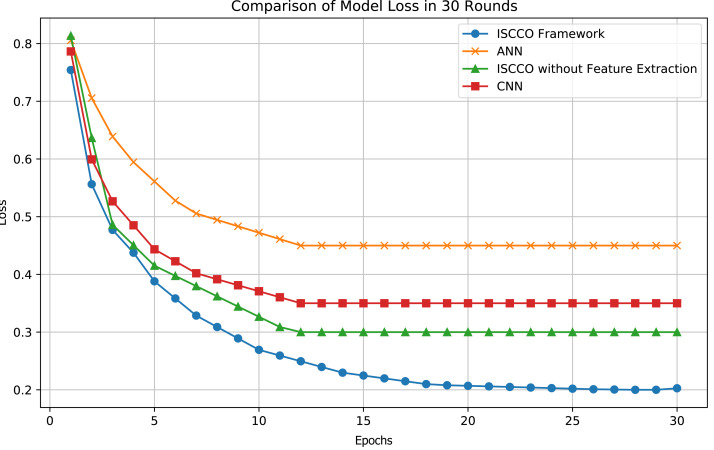
Model loss comparison.

In terms of loss values, the ISCCO framework rapidly decreased from 0.75 to 0.2, demonstrating its excellent learning efficiency and optimization capabilities. By comparison, the ANN model reduced from 0.8 to 0.45, with neither the magnitude nor the speed of loss reduction matching that of the ISCCO framework. The convolutional neural network (CNN) model performed better than the ANN but still fell short of the ISCCO framework. These data fully showcase the comprehensive advantages of the ISCCO framework in feature extraction and random forest classification, particularly in handling complex data structures and maintaining model stability, as shown in Fig. 7.

As shown in Table 4, the ISCCO framework excels in all indicators. This reflects the powerful effects of integrating autoencoder feature extraction with the random forest algorithm. Particularly in comparison to the ISCCO version without feature extraction, there was a significant improvement in accuracy, precision, and recall, proving the importance of feature extraction in enhancing the overall performance of the model. While the ANN and CNN also showed good performance, they still lag behind the ISCCO framework. The ANN model performed worse than the ISCCO framework across all three indicators, likely due to its lack of effective feature processing mechanisms. The CNN performed slightly better than the ANN but was still less capable than the ISCCO Framework in handling complex data structure classification tasks.

We conducted 10-fold cross-validation to ensure model robustness. The ISCCO Framework achieved an average accuracy of 95.73%, demonstrating consistent performance across different data splits. ANN and CNN models yielded average accuracies of 72.00% and 77.59%, respectively.

### Practical application results of the ISCCO framework

To comprehensively assess the impact of pre-shipment strategies on order cancellation rates and profits, this study designed a series of simulations with fixed order quantities. By randomly selecting a fixed proportion of orders from the overall order dataset as the experimental group for early shipment, we systematically analyzed and compared the specific effects of different pre-shipment proportions on operational outcomes while controlling other variables. As shown in Table 5, the ISCCO framework strategically increases the proportion of early shipments to optimize profits. As the pre-shipment proportion increases, the optimized profits generally rise. At 5% early shipment, the profit is slightly lower than the actual profit, but as the proportion increases, the optimized profits exceed the actual profits, particularly at 25% early shipment, where the profit increase is significant. This indicates that the algorithm effectively identifies which shipments benefit most from early dispatch.

Table 6 shows that under the ISCCO framework, the cancellation rate continues to decline compared to the baseline rate. The algorithm’s ability to reduce order cancellations is significant, as each prevented cancellation not only saves costs associated with handling returns but also stabilizes revenue streams. By predicting and managing cancellation risks, the ISCCO framework enhances customer satisfaction and retention by ensuring timely product delivery.

### Discussion

This study focuses on minimizing transportation cost losses, particularly through customer segmentation and optimized early goods allocation to reduce costs associated with order cancellations. The experimental results further validate the effectiveness of these strategies. As shown in Fig. 6 and Table 4, the ISCCO framework significantly outperforms other models in terms of accuracy, precision, and recall, achieving a notable improvement from 50% to 95.73% in classification accuracy. This improvement demonstrates the crucial role of feature extraction combined with random forest classification in customer segmentation and prediction accuracy.

(1) Our analysis revealed a strong relationship between sensitivity to discounts and cancellation behavior across different customer groups. Customers with higher price sensitivity, especially in e-commerce and fast-moving consumer goods industries, showed a greater likelihood of order cancellations, particularly during periods of economic instability. These findings are supported by the superior performance of the ISCCO framework in accurately classifying such customer segments. As the classification results highlight, businesses must adopt flexible pricing strategies and adjust resource allocations dynamically to mitigate potential cost losses due to cancellations.

**Table 4:** Detailed performance comparison of models for customer classification task.

**Model name**	**Accuracy (%)**	**Precision (%)**	**Recall (%)**
ANN	72.00	70.29	68.67
CNN	77.59	75.21	73.97
ISCCO without Feature Extraction	86.00	83.15	81.83
ISCCO Framework	95.73	93.34	90.45

**Table 5:** Profits generated by the ISCCO framework in practical application through allocation strategy.

Early shipment (%)	Actual profit	ISCCO optimized profit	Improvement
5	$114,476.38	$114,311.24	−$165.14
10	$113,753.07	$115,079.72	$1,326.65
15	$114,648.36	$117,094.41	$2,446.05
20	$115,645.46	$118,384.11	$2,738.65
25	$113,643.84	$121,204.11	$7,560.27

**Table 6:** Probability of order cancellation in practical application through allocation strategy by the ISCCO framework.

Early shipment (%)	Base cancellation rate (%)	ISCCO optimized cancellation rate (%)
5	57.93	52.43
10	56.93	49.67
15	55.04	46.91
20	54.67	44.15
25	50.79	41.39

(2) For customer groups with higher cancellation rates, our simulations (refer to Table 6) indicate that optimized early shipments, guided by the ISCCO framework, significantly reduce cancellation rates, resulting in improved operational efficiency and profit margins. The ISCCO framework’s capacity to identify optimal pre-shipment strategies underscores its potential for practical applications in reducing cancellations and enhancing overall profitability.

(3) Although the ISCCO framework performs well in reducing transportation cost losses, its limitations should not be overlooked. The quality of data is crucial to the model’s performance, and incomplete or inaccurate data can affect the accuracy of predictions. The framework focuses on reducing order cancellations and is limited in addressing issues such as inventory shortages or sudden demand spikes. Future work could consider expanding the model to tackle a wider range of logistical challenges.

In conclusion, the experimental data support the hypothesis that customer segmentation based on sensitivity to discounts and cancellation behavior, coupled with optimized early allocation strategies, plays a key role in minimizing transportation cost losses and improving market competitiveness.

## Conclusion

The ISCCO framework proposed in this study effectively integrates deep learning and optimization algorithms, significantly enhancing the efficiency and accuracy of goods allocation within the supply chain. Through an in-depth analysis of large-scale supply chain data, we have demonstrated the effectiveness of the predictive model in reducing order cancellations and optimizing transportation costs. The customer classification strategy, which combines autoencoders with random forests, supports precise goods allocation decisions, resulting in significant cost reduction and enhanced service efficiency. Experimental results indicate that the pre-shipment strategy effectively reduces order cancellation rates, thereby increasing overall profits and customer satisfaction. Additionally, the ISCCO framework exhibits low time and space complexity when handling large datasets, making it suitable for deployment in real-world complex environments.

##  Supplemental Information

10.7717/peerj-cs.2537/supp-1 Supplemental Information 1 ISCCO

10.7717/peerj-cs.2537/supp-2 Supplemental Information 2 Result

## Appendix: Mathematical Theorems and Corollary Proofs

Theorem 1 Customer classification optimization *By integrating autoencoder and random forest methods, the classification accuracy across various customer dimensions can be significantly improved. There exists an optimal set of parameters Θ^∗^, *W*^∗^, *b*^∗^ that maximizes the overall classification performance of the model:*

(28) \begin{eqnarray*}{\Theta }^{\ast },{W}^{\ast },{b}^{\ast }=\arg \nolimits \min _{\Theta ,W,b} \left\{ J(\Theta ,W,b)-\lambda \cdot \sum _{i=1}^{N}{\alpha }_{i}\cdot \log \nolimits \frac{p({y}_{i}{|}\Theta ,W,b,{x}_{i})}{p({y}_{i}{|}{x}_{i})} \right\} \end{eqnarray*}

*Here, *J*(Θ, *W*, *b*) is the model performance evaluation function based on cross-validation, *λ* and *α*_*i*_ are adjustment coefficients, where *α*_*i*_ represents the importance of the *i*-th data point, *p*(*y*_*i*_|*x*_*i*_) is the probability of category *y*_*i*_ given input *x*_*i*_.*

Proof 1 *Consider a model that integrates an autoencoder with a random forest for classifying customers in a multidimensional feature space. The autoencoder component is responsible for extracting useful nonlinear features from high-dimensional data, while the random forest component is used for classification and prediction. Each Metric_*i*_ represents a performance metric used to evaluate the model, such as accuracy, recall, F1-score, etc. Its definition is as follows:*

(29) \begin{eqnarray*}{\text{Metric}}_{i}(M,D)=\text{Specific Measurement Method}(M,D)\end{eqnarray*}

*where *M* denotes the model, and *D* represents the dataset. During the *j*-th fold of cross-validation, the performance of model *M*_*j*_ on the corresponding validation set *D*_val,*j*_ is calculated using Metric_*i*_:*

(30) \begin{eqnarray*}{\text{Performance}}_{ij}={\text{Metric}}_{i}({M}_{j},{D}_{\text{val},j})\end{eqnarray*}

*For each evaluation metric *i*, the average performance over all *k* folds is computed:*

(31) \begin{eqnarray*}{\text{Average Performance}}_{i}= \frac{1}{k} \sum _{j=1}^{k}{\text{Performance}}_{ij}\end{eqnarray*}

*This reflects the model’s average performance across different validation sets under that metric. The overall performance *J*(Θ, *W*, *b*) is the weighted average of all metrics, with weights determined by *α*_*i*_:*

(32) \begin{eqnarray*}J(\Theta ,W,b)=\sum _{i=1}^{n}{\alpha }_{i}\cdot {\text{Average Performance}}_{i}\end{eqnarray*}

*where *α*_*i*_ reflects the importance of each performance metric. *J* explicitly depends on the model parameters Θ, *W*, *b* since each fold’s model *M*_*j*_ is trained using these parameters. This dependency highlights the impact of different parameter configurations on model performance. First, define the performance evaluation function *J* to measure the model’s performance across different cross-validation sets as follows:*

(33) \begin{eqnarray*}J(\Theta ,W,b)=\sum _{i=1}^{n}{\alpha }_{i} \left( \frac{1}{k} \sum _{j=1}^{k}{\text{Metric}}_{i}({M}_{j},{D}_{\text{val},j}). \right) \end{eqnarray*}

*Here, *α*_*i*_ represents the weight of each performance metric, *M*_*j*_ denotes the model obtained from the *j*-th fold cross-validation, *D*_val,*j*_ is the corresponding validation dataset, and Metric_*i*_ is the performance evaluation metric. Next, introduce a regularization term based on Bayesian information criterion, aimed at balancing model fit and complexity. We define the conditional probability *p*(*y*_*i*_|Θ, *W*, *b*, *x*_*i*_) and the marginal probability *p*(*y*_*i*_|*x*_*i*_):*

(34) \begin{eqnarray*}p({y}_{i}{|}\Theta ,W,b,{x}_{i})=Probability~of~predicting~({y}_{i})~given~the~model~parameters~and~input~({x}_{i})\end{eqnarray*}

(35) \begin{eqnarray*}p({y}_{i}{|}{x}_{i})=Marginal~probability~of~({y}_{i})~based~only~on~input~({x}_{i}),\nonumber\\\displaystyle  ~independent~of~the~model~parameters.\end{eqnarray*}

*Compute the log-likelihood ratio, a measure of how the model provides better predictions relative to a simple probability model (dependent only on the input data):*

(36) \begin{eqnarray*}\log \nolimits \frac{p({y}_{i}{|}\Theta ,W,b,{x}_{i})}{p({y}_{i}{|}{x}_{i})} \end{eqnarray*}

*The logarithm of this ratio represents the information gain or loss between model predictions and simple predictions. Multiply each data point’s log-likelihood ratio by weight *α*_*i*_, reflecting the importance of different data points in model training:*

(37) \begin{eqnarray*}{\alpha }_{i}\cdot \log \nolimits \frac{p({y}_{i}{|}\Theta ,W,b,{x}_{i})}{p({y}_{i}{|}{x}_{i})} \end{eqnarray*}

*Sum the weighted log-likelihood ratios of all data points to form an overall regularization term:*

(38) \begin{eqnarray*}\sum _{i=1}^{N}{\alpha }_{i}\cdot \log \nolimits \frac{p({y}_{i}{|}\Theta ,W,b,{x}_{i})}{p({y}_{i}{|}{x}_{i})} \end{eqnarray*}

*Multiply the overall expression by a negative regularization coefficient *λ* to get the final regularization term:*

(39) \begin{eqnarray*}-\lambda \cdot \sum _{i=1}^{N}{\alpha }_{i}\cdot \log \nolimits \frac{p({y}_{i}{|}\Theta ,W,b,{x}_{i})}{p({y}_{i}{|}{x}_{i})} \end{eqnarray*}

*This negative sign indicates that we are penalizing high log-likelihood ratios, meaning that the higher the model complexity, the greater the penalty. This helps enhance the model’s generalization ability.* *Based on the above equations, we summarize as follows:*

(40) \begin{eqnarray*}-\lambda \cdot \sum _{i=1}^{N}{\alpha }_{i}\cdot \log \nolimits \frac{p({y}_{i}{|}\Theta ,W,b,{x}_{i})}{p({y}_{i}{|}{x}_{i})} \end{eqnarray*}

*Here, *λ* is the regularization coefficient, *p*(*y*_*i*_|Θ, *W*, *b*, *x*_*i*_) represents the conditional probability of predicting *y*_*i*_ given the model parameters and input *x*_*i*_, and *p*(*y*_*i*_|*x*_*i*_) is the marginal probability of *y*_*i*_. Combining these components, we form the final optimization problem:*

(41) \begin{eqnarray*}{\Theta }^{\ast },{W}^{\ast },{b}^{\ast }=\arg \nolimits \min _{\Theta ,W,b} \left\{ J(\Theta ,W,b)-\lambda \cdot \sum _{i=1}^{N}{\alpha }_{i}\cdot \log \nolimits \frac{p({y}_{i}{|}\Theta ,W,b,{x}_{i})}{p({y}_{i}{|}{x}_{i})} \right\} \end{eqnarray*}

*This optimization objective aims to find parameters Θ, *W*, *b* that maximize the model’s cross-validation performance while ensuring the model does not overfit the training data, achieving optimal generalization.*

Corollary 3 Cost-effectiveness enhancement *By properly allocating goods that can be shipped ahead of schedule based on customer classification, transportation cost losses caused by canceled orders can be effectively reduced. By applying the optimized classification model, goods shipped ahead of time can be more accurately allocated to users across various dimensions, thus maximizing cost-effectiveness:*

(42) \begin{eqnarray*}{\Theta }_{eff}^{\ast }=\arg \nolimits \min _{\Theta } \left\{ J(\Theta )+\gamma \cdot \text{Var}(C(\Theta )) \right\} \end{eqnarray*}

*Here, *J*(Θ) is the cross-validation performance evaluation based on the model, *γ* is a tuning factor, *Var*(*C*(Θ)) represents the variance based on the random forest model’s classification of various customer dimensions, intended to measure the stability and accuracy of classification outcomes.*

Proof *The cross-validation performance evaluation function *J* is calculated as follows:*

(43) \begin{eqnarray*}J(\Theta ,W,b)=\sum _{i=1}^{n}{\alpha }_{i} \left( \frac{1}{k} \sum _{j=1}^{k}{\text{Metric}}_{i}({M}_{j},{D}_{\text{val},j}) \right) \end{eqnarray*}

*where *α*_*i*_ are the weights of the evaluation metrics, ensuring that performance across various aspects is considered. The gradient ∇*J* is the derivative of the loss function *J* with respect to the parameters Θ, indicating the direction of steepest increase in the parameter space:*

(44) \begin{eqnarray*}\nabla J({\Theta }_{old},{W}_{old},{b}_{old})={ \left( \frac{\partial J}{\partial \Theta } , \frac{\partial J}{\partial W} , \frac{\partial J}{\partial b} \right) }_{\text{at}~({\Theta }_{old},{W}_{old},{b}_{old})}\end{eqnarray*}

*Model parameters are adjusted through the following optimization step:*

(45) \begin{eqnarray*}{\Theta }_{new}={\Theta }_{old}-\eta \nabla J({\Theta }_{old},{W}_{old},{b}_{old})\end{eqnarray*}

*η is the learning rate, determining the step size for parameter updates. Through this parameter optimization process, we expect Θ^∗^, *W*^∗^, *b*^∗^ to effectively reduce the objective function value, thereby enhancing the model’s accuracy and generalization capabilities. The inference focuses on how the optimized model can reduce transportation costs by properly allocating goods based on classification:*

(46) \begin{eqnarray*}{\Theta }_{eff}^{\ast }=\arg \nolimits \min _{\Theta } \left\{ J(\Theta )+\gamma \cdot \text{Var}(C(\Theta )) \right\} \end{eqnarray*}

*γ is a tuning factor, and Var(*C*(Θ)) represents the variance of classification outcomes, used to evaluate the stability and accuracy of classification.*

Theorem 4 Optimizing integer linear programming with parallel genetic algorithm *There exists an optimal set of parameters Θ^∗^ that optimizes the model under given cost functions and constraints:*

(47) \begin{eqnarray*}{\Theta }^{\ast }=\arg \nolimits \min _{\Theta } \left\{ Z(\Theta ,x,y)+\beta \cdot Var(x,y)+\gamma \cdot Stab(x,y) \right\} \end{eqnarray*}

*Where *Z*(Θ, *x*, *y*) is the cost function, *Var*(*x*, *y*) represents the variance of the solution, *Stab*(*x*, *y*) is the stability of the solution.*

Proof *The cost function *Z*(Θ, *x*, *y*) represents the total cost in an integer linear programming problem, which includes costs due to order cancellations, delivery delays, and storage. We will explain and verify this cost function step by step with the following equations. The cost of order cancellations and non-cancellations are expressed as:*

(48) \begin{eqnarray*}{Z}_{\text{cancel}}(\Theta ,x,y)=\sum _{i=1}^{n}\sum _{j=1}^{m}{c}_{ij}\cdot {x}_{ij}\end{eqnarray*}

*where *c*_*ij*_ is the cost incurred when item *j* in order *i* is cancelled, and *x*_*ij*_ is a binary decision variable indicating whether item *j* is assigned to order *i*. The calculation of penalties for delayed shipments is as follows:*

(49) \begin{eqnarray*}{Z}_{\text{delay}}(\Theta ,x,y)=\sum _{i=1}^{n}\sum _{j=1}^{m}{p}_{ij}\cdot (1-{x}_{ij})\end{eqnarray*}

*where *p*_*ij*_ is the penalty if item *j* in order *i* is delayed. Next, we compute the storage cost:*

(50) \begin{eqnarray*}{Z}_{\text{storage}}(\Theta ,x,y)=\lambda \sum _{i=1}^{n}\sum _{j=1}^{m}{s}_{ij}\cdot {x}_{ij}\end{eqnarray*}

*where *s*_*ij*_ is the cost of storing or keeping item *j* in order *i*, and *λ* is a cost adjustment factor that adjusts the weight of storage costs in the total cost. Then, integrating all cost components:*

(51) \begin{eqnarray*}Z(\Theta ,x,y)={Z}_{\text{cancel}}(\Theta ,x,y)+{Z}_{\text{delay}}(\Theta ,x,y)+{Z}_{\text{storage}}(\Theta ,x,y)\end{eqnarray*}

*This equation consolidates all costs arising from order cancellations, delays, and storage. It calculates the total cost by taking into account the costs of order cancellations, delay penalties, and storage. Thus, the cost function expression is:*

(52) \begin{eqnarray*}Z(\Theta ,x,y)=\sum _{i=1}^{n}\sum _{j=1}^{m} \left( {c}_{ij}\cdot {x}_{ij}+{p}_{ij}\cdot (1-{x}_{ij}) \right) +\lambda \sum _{i=1}^{n}\sum _{j=1}^{m}{s}_{ij}\cdot {x}_{ij}\end{eqnarray*}

*The cost function *Z* includes the direct costs of item cancellations and delays *c*_*ij*_ and *p*_*ij*_, as well as storage costs *s*_*ij*_.*

Corollary 4 Parameter tuning and system performance enhancement *In the integer linear programming model enhanced with a parallel genetic algorithm, optimizing parameters Θ can achieve higher system performance and cost-effectiveness:*

(53) \begin{eqnarray*}{\Theta }^{\ast }=\arg \nolimits \min _{\Theta } \left\{ \alpha \cdot Z(\Theta )+\beta \cdot Var(C(\Theta ))+\gamma \cdot Stab(C(\Theta )) \right\} \end{eqnarray*}

*Here, *α*, *β*, and *γ* are weight factors used to balance cost, variance, and stability.*

Proof *The optimization problem is defined as finding a set of parameters Θ that minimize the given composite objective function. This objective function combines the cost function *Z*, the variance of solutions Var, and stability Stab. The definition of variance Var(*x*, *y*) is:*

(54) \begin{eqnarray*}\text{Var}(x,y)= \frac{1}{N} \sum _{i=1}^{N}({y}_{i}-\mu )^{2}\end{eqnarray*}

*where *y*_*i*_ represents a specific metric of the *i*th solution, and *μ* is the average of all solutions. Variance is used to evaluate the diversity of the solution set. The definition of stability Stab(*x*, *y*) is:*

(55) \begin{eqnarray*}\text{Stab}(x,y)=1- \frac{\text{Var}(x,y)}{{\sigma }^{2}} \end{eqnarray*}

*where *σ*^2^ is the possible maximum variance, expressing the stability of the solution, *i.e.,* the consistency of the solution under different runs or conditions. The composite objective function is:*

(56) \begin{eqnarray*}F(\Theta ,x,y)=Z(\Theta ,x,y)+\beta \cdot \text{Var}(x,y)+\gamma \cdot \text{Stab}(x,y).\end{eqnarray*}

*This formula combines costs, diversity of solutions, and stability. By adjusting the weights *β* and *γ*, it is possible to balance the importance of these metrics. The ultimate goal is to find the optimal parameter set Θ^∗^:*

(57) \begin{eqnarray*}{\Theta }^{\ast }=\arg \nolimits \min _{\Theta } \left\{ F(\Theta ,x,y) \right\} .\end{eqnarray*}

*This equation expresses the optimization goal to find the parameter set Θ that minimizes the composite objective function *F*, ensuring the lowest cost while maintaining the diversity and stability of the solutions. The objective function for a genetic algorithm:*

(58) \begin{eqnarray*}{\Theta }^{\ast }=\arg \nolimits \min _{\Theta } \left\{ Z(\Theta ,x,y)+\beta \cdot \text{Var}(x,y)+\gamma \cdot \text{Stab}(x,y) \right\} .\end{eqnarray*}

*Here, Var(*x*, *y*) measures the diversity of solutions, and Stab(*x*, *y*) measures the stability of solutions. The mathematical expression for variance:*

(59) \begin{eqnarray*}\text{Var}(x,y)= \frac{1}{n} \sum _{i=1}^{n}{ \left( {x}_{i}-\bar {x} \right) }^{2}+ \frac{1}{m} \sum _{j=1}^{m}{ \left( {y}_{j}-\bar {y} \right) }^{2}\end{eqnarray*}

*where $\bar {x}$ and $\bar {y}$ are the means of *x* and *y* respectively. Subsequently, the calculation for stability:*

(60) \begin{eqnarray*}\text{Stab}(x,y)=1- \frac{\text{Var}(x,y)}{{\sigma }^{2}} \end{eqnarray*}

*where *σ*^2^ represents the maximum possible variance. The selection, crossover, and mutation operations of a genetic algorithm:*

(61) \begin{eqnarray*}{g}_{k}(x,y)\leq 0, {h}_{l}(x,y)=0\end{eqnarray*}

*These are the constraints of the algorithm, ensuring the feasibility of the solutions. Balancing the weight factors:*

(62) \begin{eqnarray*}\alpha \cdot Z(\Theta )+\beta \cdot \text{Var}(x)+\gamma \cdot \text{Stab}(x)\end{eqnarray*}

*By adjusting *α*, *β*, and *γ* to find the optimal solution. Minimizing the objective function:*

(63) \begin{eqnarray*}{\Theta }^{\ast }=\arg \nolimits \min _{x} \left\{ \alpha \cdot Z(\Theta ,x)+\beta \cdot \text{Var}(x)+\gamma \cdot \text{Stab}(x)+\delta \cdot \text{Cons}(x) \right\} \end{eqnarray*}

*Here, *δ* controls the degree of constraint satisfaction.Calculation of constraint satisfaction:*

(64) \begin{eqnarray*}\text{Cons}(x)=\sum _{i}\max \nolimits (0,{c}_{i}(x))\end{eqnarray*}

*This represents the sum of all unsatisfied constraints. Verification of systemic performance improvement:*

(65) \begin{eqnarray*}{\Theta }^{\ast }=\arg \nolimits \min _{\Theta } \left\{ \alpha \cdot Z(\Theta )+\beta \cdot \text{Var}(C(\Theta ))+\gamma \cdot \text{Stab}(C(\Theta )) \right\} \end{eqnarray*}

*Optimizing these parameters can enhance system performance and cost-effectiveness. The multi-objective optimization problem:*

(66) \begin{eqnarray*} & \min \nolimits  Z(\Theta ,x,y) & & \text{subject to} {g}_{k}(x,y)\leq 0, {h}_{l}(x,y)=0\end{eqnarray*}

*This ensures multi-objective and multi-constraint management of the solution. Balancing and trade-offs during the optimization process:*

(67) \begin{eqnarray*}\text{GA-ILP}(\Theta ,x)=\arg \nolimits \min _{x} \left\{ \alpha \cdot Z(\Theta ,x)+\beta \cdot \text{Var}(x)+\gamma \cdot \text{Stab}(x) \right\} \end{eqnarray*}

*This expresses how to balance costs, variance, and stability. The combination of integer linear programming and genetic algorithms:*

(68) \begin{eqnarray*}{x}_{ij}\in \{ 0,1\} , {y}_{ij}={x}_{ij}\times (1-{e}^{-\lambda {t}_{ij}})\end{eqnarray*}

*This reflects the integer nature of the decision variables and the dynamic adjustment of fulfillment probabilities.*

## Additional Information and Declarations

### Data Availability

The following information was supplied regarding data availability: The raw data is available at Alibaba Cloud and Zenodo: - https://tianchi.aliyun.com/dataset/89959
- Yangyan, L. (2024). DataCoSupplyChainDataset [Data set]. Zenodo. https://doi.org/10.5281/zenodo.11112645.
